# Microextraction Techniques Used in the Procedures for Determining Organomercury and Organotin Compounds in Environmental Samples

**DOI:** 10.3390/molecules19067581

**Published:** 2014-06-06

**Authors:** Małgorzata Rutkowska, Kinga Dubalska, Piotr Konieczka, Jacek Namieśnik

**Affiliations:** Department of Analytical Chemistry, Faculty of Chemistry, Gdańsk University of Technology, G. Narutowicza 11/13 Street, 80-233 Gdańsk, Poland; E-Mails: malgorzatahelenachmiel@gmail.com (M.R.); kinga.dubalska@gmail.com (K.D.); piotr.konieczka@pg.gda.pl (P.K.)

**Keywords:** green analytical chemistry, microextraction techniques, speciation, sample preparation, organometallic species, environmental samples, environmental analytics

## Abstract

Due to human activities, the concentrations of organometallic compounds in all parts of the environment have increased in recent decades. The toxicity and some biochemical properties of mercury and tin present in the environment depend on the concentration and chemical form of these two elements. The ever-increasing demand for determining compounds at very low concentration levels in samples with complex matrices requires the elimination of interfering substances, the reduction of the final extract volume, and analyte enrichment in order to employ a detection technique, which is characterised by high sensitivity at low limits of quantification. On the other hand, in accordance with current trends, the analytical procedures should aim at the miniaturisation and simplification of the sample preparation step. In the near future, more importance will be given to the fulfilment of the requirements of Green Chemistry and Green Analytical Chemistry in order to reduce the intensity of anthropogenic activities related to analytical laboratories. In this case, one can consider the use of solvent-free/solvent-less techniques for sample preparation and microextraction techniques, because the use of the latter leads to lowering the quantity of reagents used (including solvents) due to the reduction of the scale of analysis. This paper presents an overview of microextraction techniques (SPME and LPME) used in the procedures for determining different chemical forms of mercury and tin.

## 1. Introduction

The strive for the accurate assessment of individual elements of the environment and processes occurring in them is the driving force for the development of appropriate analytical tools, which are necessary for obtaining reliable information. This concerns:

- control and measuring devices, which ensure the possibility of analysing the prepared samples;
- reference materials which accurately reflect the composition and character of the actual samples tested, which is necessary to ensure a proper system for the quality control and assurance of measurement results;
- analytical methodologies which can be used in testing environmental samples often characterised by a complex matrix composition and low, and sometimes very low, analyte content levels.

In the latter case, the stage of preparing samples for analysis is a significant element of analytical procedures. Within this stage, three basic tasks are implemented:

○ increasing the level of analyte concentrations in the analysed samples to a higher level than the limit of detection of the analytical technique used;
○ removal of at least a part of interferents which can influence the result of the analysis;
○ replacement or at least simplification of the matrix composition of samples for analysis.

At this stage, meeting the requirements of Green Chemistry and Green Analytical Chemistry will become more and more important to make it possible to decrease the intensity of the impact of anthropopresion connected with the operations of analytical laboratories. In this case, the following options can be considered:

- the use of solvent-free/solvent-loss techniques for preparing samples for analysis;
- the use of microextraction techniques which, due to a reduced scale of the analysis lead to a decrease in the quantity of reagents used, including solvents.

This relatively broad introduction with a general description of the existing knowledge is appropriate also for organomercury and organotin compounds present in environmental samples. In this study, literature information on microextraction techniques (SPME, LPME) will be presented in procedures for the determination of various chemical forms of mercury and tin, their short description and possibility of using them in environmental research.

## 2. Analysis of Organomercury and Organotin Compounds

Mercury and tin are considered to belong to the most toxic heavy metals due to their ability to accumulate, and their permanent character in individual elements of the environment [1]. In addition, these metals occur in the environment in many physical and chemical forms [2].

**Figure 1:**
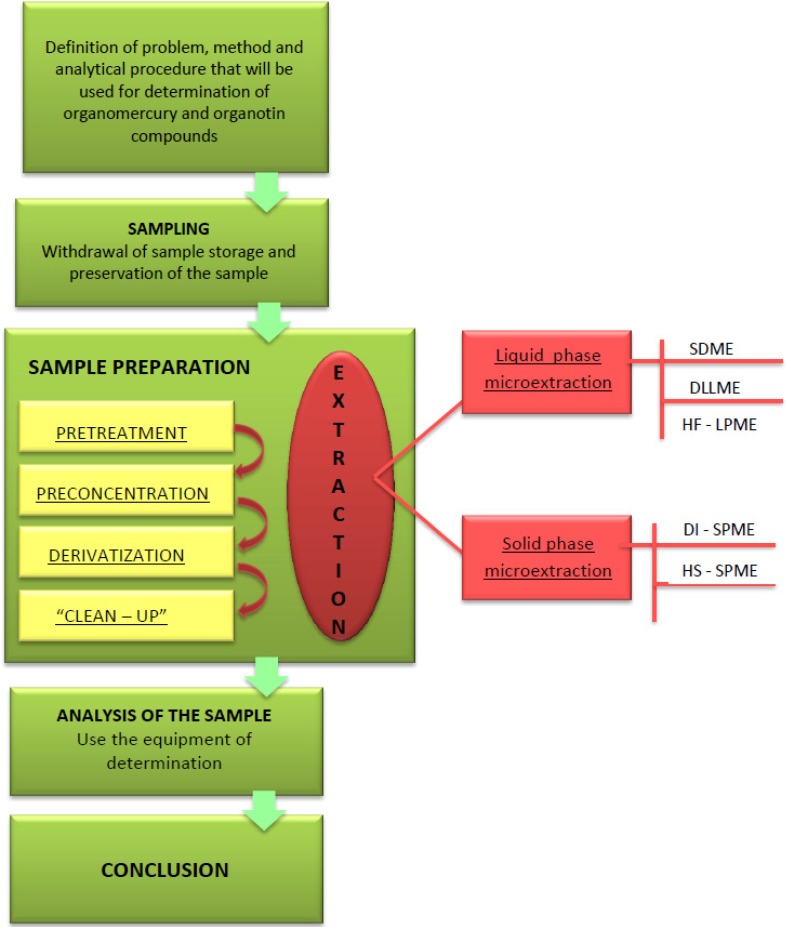
Diagram depicting the course of various procedures for determining organomercury and organotin compounds using microextraction techniques at the stage of preparing samples for analysis. Based on Oliveira, R. *et al*. [5].

The sample preparation stage in the analytical procedure for determining organomercury and organotin compounds usually involves the extraction process, which leads to the isolation and enrichment of analytes from the samples. The enrichment process is necessary due to very low levels of the content of various forms of mercury and tin in environmental samples [3]. Figure 1 shows a diagram depicting the course of various procedures for determining organomercury and organotin compounds in which microextraction techniques are used at the stage of preparing samples for analysis.

Extraction may differ in the selectivity level, the execution rate and convenience, and it does not only depend on the method and conditions, but also on the geometric configuration of the extraction phase [2].

## 3. Analytical Procedures Using Microextraction Techniques for the Liquid Phase

Classical liquid–liquid extraction (LLE) is one of the oldest techniques of enrichment and isolation in analytical chemistry and is still used in numerous analytical procedures [4]. However, while using the tedious and time-consuming LLE technique, large quantities of expensive and dangerous organic solvents are used. As a result, the latest research trends include miniaturisation of the traditional liquid–liquid extraction system, which is mostly aimed at decreasing the volumetric ratio of the acceptor-to-donor phase. Table 1 presents information on the stages of development and improvement of the microextraction technique to the liquid phase.

**Figure 2:**
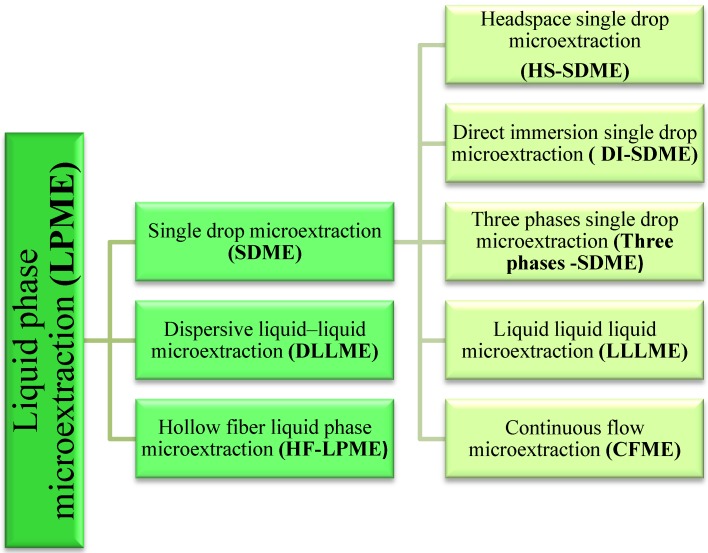
Classification of various approaches in liquid-phase microextraction.

At present, the LPME technique is used both for removing interferents, enriching analytes present in samples, and for the simplification of the composition of their matrix. The method of classification of various approaches in liquid-phase microextraction is presented in Figure 2.

**Table 1:** Stages of the development and improvement of the LPME technique [6].

Year	Methodological Solution
**1995**	First single-drop-based extraction systems
**1996**	First drop-in-drop system
**1997**	Liquid stage microextraction in a dynamic system The use of microsyringe for supporting the drop
**1999**	Liquid-phase microextraction using fibre (LPME)
**2001**	Headspace Solid-Phase Microextraction (HS-SDME)
**2003**	Using ionic liquids as the extracting agent
**2005**	Using water as a solvent in liquid-phase microextraction
**2006**	Liquid-phase microextraction using ultrasound as a factor supporting the extraction process
**2007**	Liquid-phase microextraction using microwave radiation as a factor supporting the extraction process Automation of the single-drop microextraction process
**2008**	Combining microextraction to the liquid phase with flame atomic absorption spectroscopy
**2009**	Liquid-phase microextraction using an ionic liquid combined with dispensing a sample to the column using a thermal desorption device

Liquid-phase microextraction, in which the extraction solvent has the form of one drop, is called single-drop microextraction. In this technique, the use of an organic solvent is minimised to one drop (1–8 µL), which makes it exceptionally environmentally friendly [7,8,9]. The SDME method can be used for liquid and gaseous samples. This method is an appropriate strategy for enriching the matrix composition before detection and is regarded as the basic LLME technique, which is successfully used for the extraction of organomercury and organotin compounds, e.g., from water samples [10,11].

A single solvent or a mixture of solvents can be used for extraction to obtain higher selectivity. For metal ion extraction, chelating reagents dissolved in an organic solvent are used [12].

**Figure 3:**
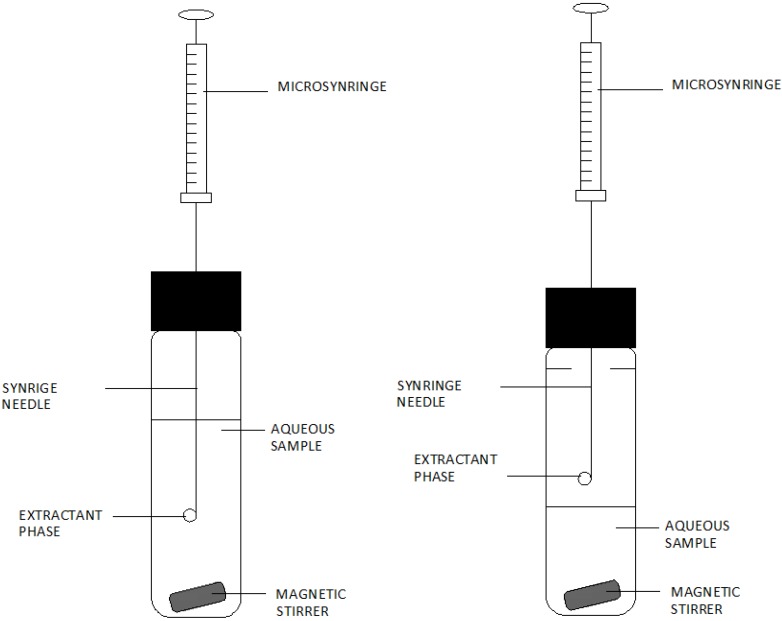
Diagram of a set for direct immersion single-drop microextraction (DI-SDME) and headspace single-drop microextraction (HSSDME).Based on Pena-Pereira, F. *et al.* [14].

In the case of the SDME technique, it is the ability to keep a solvent drop at the end of the needle, which is immersed in the analysed sample (DI) or is placed on the headspace phase of the sample (HS). Figure 3 presents this solution in the form of a diagram. Xenobiotics are divided between the sample and the organic phase based on passive diffusion [13,14].

In the case of the DI-SDME techniques, two liquid phases are in direct contact between each other, and the transfer of analytes from the water solution to the extraction drop lasts until thermodynamic balance is achieved or the extraction is stopped [11]. DI-SDME requires the use of a mixing organic solvent and analytes, which are characterised by higher solubility in the organic solvent than in the sample solution [11,13].

In the HS-SDME technique, gaseous analytes from the liquid phase, which are in the gaseous phase, dissolve in the solvent drop at the end of the microsyringe needle placed over the surface of the sample. After the extraction, lasting for a defined period of time, the microdrop is withdrawn back into the syringe needle and then it is injected to the detector or chromatograph for quantitative determination of analytes [11].

The methodological solutions described above should be treated as a static variant of this technique. Dynamic variants are also known and, in this case, a small liquid column which is in the syringe throughout the extraction is the drop [6]; the sample is then introduced into the syringe, where analytes are dissolved at the solvent phase.

Determining the different forms of mercury and tin in the water samples is troublesome due to the fact that the concentration of organometallic compounds in water is relatively low. Therefore, there is more and more information on the possibility of using the liquid–liquid–liquid microextraction technique (LLLE) to enrich analytes and purify extracts for the analysis of organomercury and compounds in water samples and other environmental samples.

**Figure 4:**
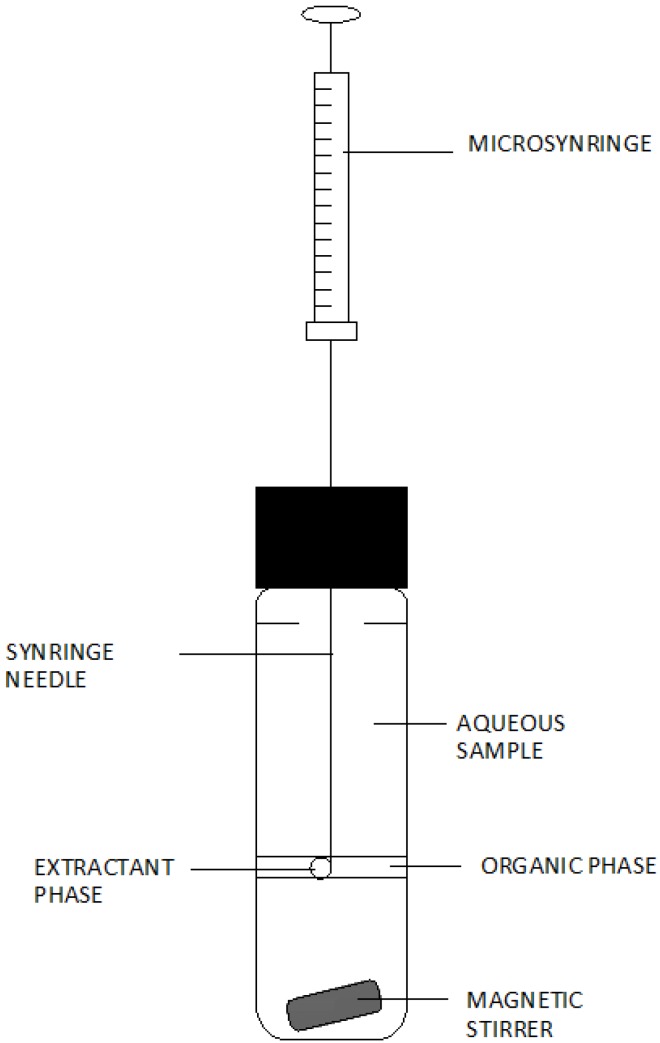
A diagram of the set for liquid–liquid–liquid microextraction. Based on Pena-Pereira, F. *et al.* [14].

The LLLME technique uses three solutions—the donor solution, the organic solvent phase, which constitutes a specific organic membrane separating the two aqueous phases, and the acceptor solution [15,16,17,18]. In general, the principle of operation in this system can be presented in the following way: analytes are extracted from the starting solution to the organic solvent phase and then it is extracted again to drops of the acceptor aqueous solution suspended in an organic solvent, usually at the end of a syringe needle. A diagram of a set for performing tests using this technique is presented in Figure 4. (In this system, the analytes are extracted from the donor solution into the organic solvent phase and back-extracted simultaneously into the acceptor phase while stirring) [15,17,18,19].

The liquid-liquid-liquid microextraction is becoming more and more popular as a technique for analyte enrichment and purification of samples containing organometallic compounds prior to analysis, in particular using capillary electrophoresis [15]. Literature contains information about numerous modifications which have been introduced into the LLLME system over the past several years. For example, the use of hollow-fibre liquid-liquid-liquid microextraction for the determination of organomercury compounds [16,20,21,22].

**Figure 5:**
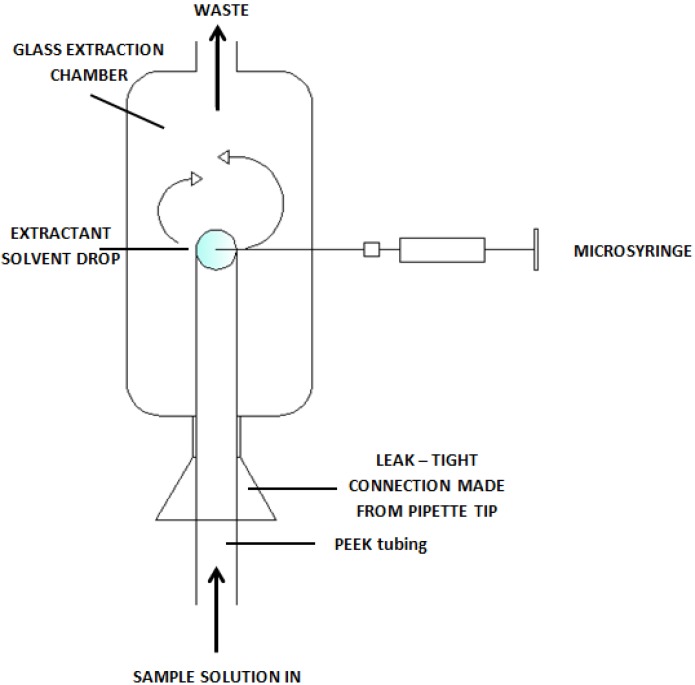
A diagram of a set for continuous flow microextraction [23].

For instance various techniques of simple microextraction to the liquid phase were improved to eliminate some defects of the traditional installations. In 2000, the principles of the continuous-flow microextraction technique (CFME) were first described. In this technique, a drop of extractant solvent is introduced using a microsyringe into the extraction chamber so as to place it on the outlet tip of the PEEK tube. This tube is used as a kind of “holder” for a solvent drop and for filling the extraction chamber by pumping the sample through it in a continuous manner at a constant flow rate. As a result, the solvent drop placed at the end of the tube has continuous contact with the sample solution. At the end of the extraction process, the extract is collected using a microsyringe [23]. Figure 5 presents a diagram of the construction of the set for tests using the CFME technique.

**Figure 6:**
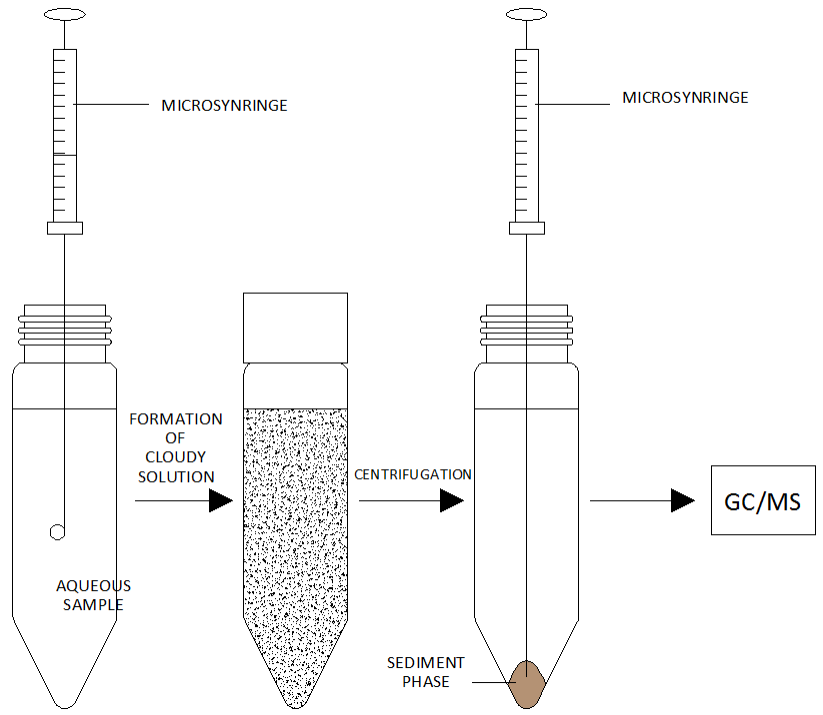
A diagram presenting consecutive stages of dispersive liquid–liquid microextraction.Based on Pena-Pereira, F. *et al.* [6].

The dispersive liquid–liquid microextraction (DLLME) is a relatively “young” extraction technique, developed in 2006 [3]. This technique was initially used for isolating and enriching organic compounds, such as polycyclic aromatic hydrocarbons (PAH), organophosphate pesticides and chlorobenzenes [24]. It was also used in procedures for determining organotin compounds and other inorganic compounds. The DLLME technique is based on the use of a triple solvent system, just like in homogeneous liquid-liquid extraction (LLE) and cloud point extraction (CPE). Dispersive liquid-liquid microextraction consists of two stages (Figure 6):

(a) The introduction of an appropriate extraction and dispersing solvent mixture into an aqueous solution of an analyte-containing sample.

The quantity of the extraction solvent used is usually approx. 1%–3% of the total mixture volume of various extraction solvents. At this stage, the extraction solvent is dispersed in the aqueous sample in the form of fine drops, in which analytes are enriched. At this stage, the solution becomes cloudy [13]. The state of balance is achieved quickly due to the large surface area between the extraction solvent and the aqueous sample solvent so that the extraction hardly depends on time.

(b) Centrifugation of the cloudy solution.

After the completion of the microextraction process, centrifugation is a necessary step to separate the extracting agent phase. The enriched extracting agent phase is used to determine the required analytes using conventional analytical techniques [6,13].

The organic solvent is used on the basis of its density, which should be higher than the density of water, the extraction ability towards the analytes and compatibility with the chromatographic system used. Usually, these are: chlorobenzene, chloroform, carbon tetrachloride or tetrachloroethylene. The dispersing solvent must mix with water and the polar solvent. Acetone, methanol and acetonitrile are usually used as dispersing solvents [11,13].

At present, this technique is mostly used for analysing simple water samples and for preliminary tests. An additional purification stage would be required for samples characterised by a complex matrix composition [11,13].

**Figure 7:**
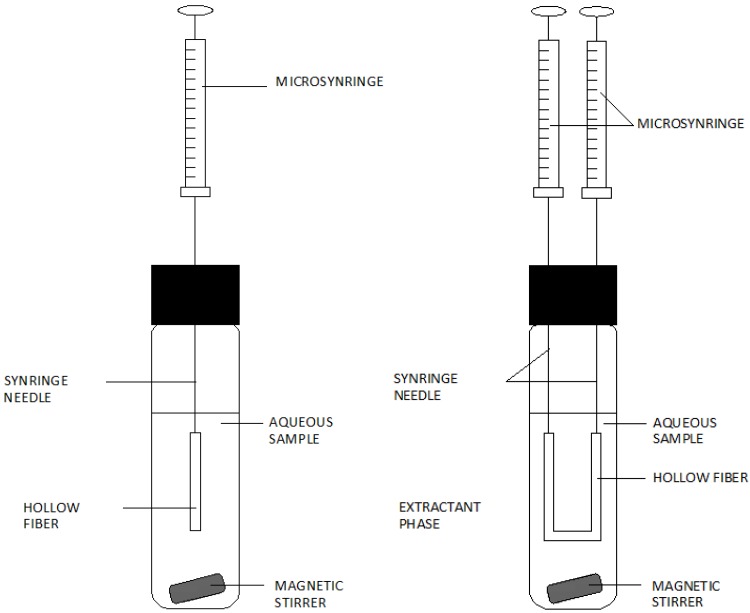
A diagram of the structure of a set for hollow-fibre liquid phase microextraction (HF-LPME).Based on Pena-Pereira, F. *et al*. [6].

Single-drop microextraction, apart from numerous significant advantages, has a disadvantage connected with the risk of destroying the drop. As a result, by modifying microextraction techniques to the liquid phase, changes were introduced, which involved immobilisation of the extracting liquid in a porous fibre drain, which was used in Hollow-fibre liquid-phase microextraction (HF-LPME) [12]. This is extraction in the liquid–liquid system, where the extracting liquid is situated in the spaces of a porous fibre fixed at the tips of two needles (Figure 7, right-hand side) or at the end of one microsyringe needle (Figure 7, left hand side). Before the commencement of the extraction process, the capillary fibre is immersed in an organic solvent to “keep” the organic solvent in the pores of the capillary fibre. The capillary fibre is then immersed in the sample solution. To accelerate the extraction, the sample is intensively shaken or mixed. After the completed extraction, the extract is drawn into a syringe from the capillary tube.

This technique gives the possibility of ensuring high repeatability and high selectivity of the extraction process due to the possibility of using a broad spectrum of appropriate extracting liquid and the type of porous fibre. Apart from protecting the extracting liquid, the fine pores of the fibre prevent weight materials from getting into it, which is desirable especially in the analysis of biological liquids. The extraction process using the HF-LPME technique can be performed both in two-phase and three-phase systems [25]. In two-phase systems, the acceptor solution is the same organic solution which was immobilised in the pores, while analytes are collected in the organic phase, which is compatible with the GC [26]. In three-phase systems, however, the acceptor solution is another aqueous phase and analytes are extracted from the aqueous sample solution into a water acceptor solution through a thin layer of organic solvent. This is an excellent solution when combined with the HPLC, CE and AS technique, at the analyte separation and determination stage [11,13,26]. Examples of applying these techniques for the liquid phase microextraction in combination with various separation techniques in organometallic compounds determination procedures are summarised in Table 2.

**Table 2:** Examples of application of selected microextraction techniques for analyte sampling before the determination of various forms of tin and mercury.

Sample Type			Species					Method						Derivatization						Fiber/Extraction Time/Extraction Mode Or Extractant Phase/Drop Volume (µL)/Sample Volume (mL)/Extraction Time (min)/Stirring Rate (Flow Rate)					Detection Technique						E.F.						Precision (RSD %)						Detection Limit						Reference			
Gas condensate			Met_2_-Hg					SPME						None (direct sampling)						100 µm PDMS/30 s/HS					MIP-AES						-						-						20 µg/L						[27]			
Water, fish tissue			MetHg					SPME						NaBEt_4_/acetate buffer pH 4.5						100 µm PDMS/5 min/HS					AFS						-						-						3.0 ng/L						[28]			
Water, seawater			TeMT TMT DMT MMT					SPME						NaBEt_4_/acetic acid buffer pH 4						100 µm PDMS/20 min/HS					FPD						-						-						41 ng/L 15 ng/L 8.4 ng/L 8.6 ng/L						[29]			
Surface water, sediment			Alkylmercury Alkyltin					SPME						NaBEt_4_/acetate buffer pH 5.0						100 µm PDMS/10 min/HS					ICP-MS						-						-						3.7 ng/L 0.38–1.2 ng/L						[30]			
Sediment			MBT DBT TBT MetHg					SPME						NaBEt_4_/acetate buffer pH 5.3						100 µm PDMS/10 min/HS					ICP-MS						-						-						0.34 ng/L 2.1 ng/L 1.1 ng/L 4.3 ng/L						[27,31]			
Sediment, sewage sludge			MBT DBT TBT MPhT DPhT TPhT					SPME						NaBEt_4_/ethanoic acid buffer pH 4.8						100 µm PDMS/60 min/LPh					FPD						-						-						0.031 ng/L 0.007 ng/L 0.006 ng/L 0.114 ng/L 0.167 ng/L 0.583 ng/L						[32]			
Slurry of sediment			MBT DBT TBT TeBT					SPME						NaBEt4/acidified with HCl						100 µm PDMS/45 min/LPh					MIP-AES						-						-						µg/L range						[33]			
Soil			MetHg EtHg PhenHg					SPME						Hydride generation (KBH_4_)/acetate buffer pH 4						Fused-silica fiber (pretreated with conc. HF acid for 3.5–4 h)/1.5–2 h/HS					AAS (quartz tube)						-						-						Not reported						[27]			
Soil			Et_2_-Hg Met_2_-Hg					SPME						None (direct sampling)						100 µm PDMS/20 min/HS					MIP-AES						-						-						3.5 µg/L						[27]			
Environmental, sediment			MBT DBT TBT					SPME						NaBEt_4_/acetate buffer pH 4						100 µm PDMS/60 min/HS					FID						-						-						10 µg/L 1.2 µg/L 0.9 µg/L						[27]			
Body fluids			MBT DBT TBT MetHg Hg^2+^					SPME						NaBEt_4_/acetate buffer pH 5.3						100 µm PDMS/10 min/HS					EI-MS-MS						-						-						9 ng/L 13 ng/L 9 ng/L 22 ng/L 18 ng/L						[34]			
Urine			MetHg Hg^2+^					SPME						NaBEt_4_/buffer pH 4						100 µm PDMS/15 min/HS					EI-MS						-						-						303 ng/L 93 ng/L						[35]			
Biological samples, sediments			MetHg					SPME						Hydride generation (KBH4)/acetate buffer pH 3						Fused-silica fiber (pretreated with conc. HF acid for 3.5–4 h)/1.5–2 h/HS					AAS (quartz tube)						-						-						Not reported						[27]			
Seawater samples, Sediment sample, Biological samples (fish, crab, prawn)			MeHg					SPME						Na[B(C6H5)4]/acetate buffer pH = 4.5						100 µm PDMS/15 min					GC-MS						-						-						0.02						[36]			
Aqueous samples			Organotin					HS-SPME						NaBEt_4_ ( *in situ*)/ammonia/citrate buffer pH 8.5						100 mm PDMS					GC-AED						-						-						pg/L ng/L						[37,38]			
			Organomercury											NaBEt_4_ ( *in situ*)/ammonia/citrate buffer pH 5						CW/PDMS																																
Natural water			MBT, TBT, MetHg Hg^2+^					HS-SPME						2% NaBEt_4_/0.2 M acetic acid and 0.2 M sodium acetate/pH 5.5						PDMS/30 min					GC-EI-MS						-						-						below ng/L or sub ng/L						[38,39]			
Marine sediments			MBT, DBT, TBT					HS-SPME						NaBEt_4_ ( *in situ*)						PDMS					GC-MS						-						-						730–969 pg/g						[37,39,40]			
Estuarine superficial sediment			MBT, DBT, TBT					HS-SPME						NaBEt_4_/1.5 M sodium acetate Buffer/pH 4.3						100 mm PDMS/15 min					GC-FID						-						-						-						[37,39,41]			
Biological materials and road dust			TMT, DMT, MMT, MBT, DBT, TBT					HS-SPME						-						PDMS/DVB					MC-GC-ICP-TOFMS						-						-						below pg/g						[37,42]			
			MetHg																	CAR/PDMS																							2 pg/g									
			Hg^2+^																	CAR/PDMS																							1.3 pg/g									
Natural water			TMT, DMT, MMT, MetHg Hg^2+^					HS-SPME						NaBEt_4_/buffer pH 5.3						PDMS µm					GC-MS						-						5 3 20 14 20						level ng/L						[39]			
																				DVB/CAR/PDMS 50 µm/30 µm/30 min/5 mL																																
Water samples			MeHg DBT TBT					HS-SPME						NaBEt_4_						100 µm PDMS/or 50 µm/30 µm DVB/CAR/PDMS 30 min for MeHg/60 min for DBT and TBT					GC-MS						-						5 14 20						3 ng/L 7 ng/L 16.8 ng/L						[39]			
Aqueous samples			MetHg Hg^2+^					DI-SPME						-						PDMS					GC-MS						-						-						-						[43]			
-			MMT DMT TMT MBT DBT TBT TPT Dioctyltin MetHg EtHg PhenHg Met_2_-Hg Et_2_-Hg					SDME						-						[C_4_MIM][PF_6_]/[C_8_MIM][PF_6_]/5/10/15 (30)/-					ETAAS						28/18 28/20 90/161 12/14 10/11 15/23 32/24 35/28 5/4 15/13 40/27 15/7 32/14						-						-						[44]			
																									CV-AFS																											
Water			Hg Sn					SDME						NaBH4 in the sample; Pd(II) in the drop						Pd(II)/3/5/3.5/1000 rpm											72 37						8.7 8.2						800 90						[45]			
Water			Hg					SDME						H2Dz in the drop						m-Xylene containing H2Dz/10/ 15/20/300 rpm					ETAAS						970						6.1						10 ng/L						[46]			
Tuna fish and dogfish muscle			MetHg					SDME						NaBH4 in the sample; Pd(II) in the drop						Pd(II)/3/5/3/300 rpm					ETAAS						40						7						5000						[47]			
-			Organotin					HS-SDME						-						Decane/11 min					GC-MS						-						3.6						TBT: 3 (Sn) ng/L						[11,14]			
Sediment CRM			MBT DBT TBT					HS-SDME						-						Decane/11 min					GC-MS						-						3.6						3 ng/L						[10,11]			
Biological, environmental samples			MBT DBT TBT					HS-SDME						-						Decane/5 min					GC-ICP-MS						-						4.4–10.1						0.8–1.8 ng/L						[11]			
-			Organomercury					D-SDME						-						[C_4_MIM][PF_6_]/15 min					CVAAS						5–40						-						-						[14]			
-			Organotin					D-SDME						-						[C_4_MIM][PF_6_]/15 min					ETAAS						10–90						-						-						[14]			
-			TBT TPT					D-SDME						-						α,α,α,-Trifluorotoluene/60 min					GC-MS-MS						140 2.9						11 10						0.36 ng/l 2.9 ng/L						[14]			
River water			Hg					D-SDME						-						Xylene/20 min					ETAAS						970						6.1						10 ng/L						[11]			
Water samples			MetHg EtHg PhenHg Hg+					D-SDME						-						[C4MIM][PF6]/20 min					HPLC						107 31 11 3						5.3 3.7 9.4 11.6						11.0 ng/L 1.6 ng/L 7.1 ng/L 22.8 ng/L						[11,14]			
-			Organotin					DLLME						-						Tetrachloromethane, ethanol/<3 min					GC-FPD						825–1036						2.3–5.9						0.2–1 ng/L						[14]			
Water samples			MBT DBT TBT					DLLME						butyltin compounds aqueous solution pH = 4.5/NaBEt4						Tetrachloromethane, methanol/20 min					GC-MS						-						17 15 9						1.7 ng/L 2.5 ng/L 5.9 ng/L						[48]			
-			Organomercury					LLLME						-						Toluene, L–cysteine/40 min					CE-UV						210–324						6.1–7.2						430–940 ng/L						[14,15]			
Water samples			MetHg EtHg PhenHg					HF-LPME						-						Toluene, Na_2_S_2_O_3_/5 min					HPLC-UV						120 215 350						8.9 6.4 6.6						3800 ng/L 700 ng/L 300 ng/L						[11]			
Human hair, sludge			MetHg					HF-LPME						-						Toluene/0 min					ETAAS						55						11						400 ng/L						[11,26]			
Human hair, fish sample, dogfish muscle CRM			MetHg EtHg PhenHg					HF-LPME						-						Bromobennzene, L–Cysteine/50 min					LVSS-CE/UV						3610 3160 4580						3.3 3.6 7.5						140 ng/L 70 ng/L 30 ng/L						[11]			
Fish CRM			MetHg					HF-LPME						-						Toluene/10 min					ETAAS						55						11						400 ng/L						[14,26]			
Human hair, sludge and dogfish muscle			MetHg					HF-LPME						-/						Toluene/4/3/10 min/1300					ETAAS						55						11						400 ng/L						[26,49]			
														thiourea in the lumen of the fibre						Toluene/thiourea 4% (m/v) in 1 mol L−1 HCl											204						13						100 ng/L									
Fish CRM			MetHg					HF-LLLME												Toluene, thiourea/10 min					ETAAS						204						13						100 ng/L						[14,26]			
Fish CRM, water			MetHg EtHg PhenHg					HF-LLLME						-						Polypropylene /toluene (octanol, CCl4)/Na2S2O3/6/3.8/25 min					HPLC-UV						120–350						6.4–8.9						3–3.8 ng/mL						[14,16,50]			
Dogfish muscle			MeHg EtHg PhHg					HF-LLLME						-						-					On-line FIDS-HPLC						120 215 350						8.9 6.4 6.6						10–25 ng/g						[16]			
Seawater sample			MeHg EtHg					HF-LLLME						-						-					LC-ICP-MS						120 215						8.9 6.4						0.03 ng/mL 0.04 ng/mL						[16]			
Fish sample			MeHg					HF-LLLME						-						-					GC-AFS						120						8.9						1.2 pg						[16]			
Fish tissues			MeHg					HF-LLLME						-						-					GC-ICP-MS						120						8.9						2.1 ng/g						[16]			

## 4. Analytical Procedures Using Microextraction Techniques for the Stationary Phase

Microextraction for the stationary phase is an alternative approach (proposed in 1990 by Arthur and Pawliszyn) for liquid–liquid extraction [27,51]. SPME is a simple and efficient technique, which eliminates the necessity of using solvents. This method still enjoys great interest as it can be used for isolating and enriching a broad range of organic compounds, including organic forms of metals in environmental samples and in other samples with a complicated composition of the matrix [39,51,52,53].

Solvent-free extraction methods can be classified according to the type of the extraction phase, which can be:

- gas;
- membrane;
- sorbent.

The solid-phase microextraction technique (SPME) is an exceptionally useful tool for mercury and tin speciation analysis. Examples of SPME technique applications in environmental analysis are summarised in Table 2. In addition, it meets the requirements of the current trend for miniaturisation of the sample preparation set and almost complete elimination of solvents from this process [52,54,55].

Depending on the placement of the fibre relative to the sample during the extraction, it can be performed: [55,56,57]:

- directly from the tested sample (DI-SPME);
- from the headspace phase (HS-SPME).

**Figure 8:**
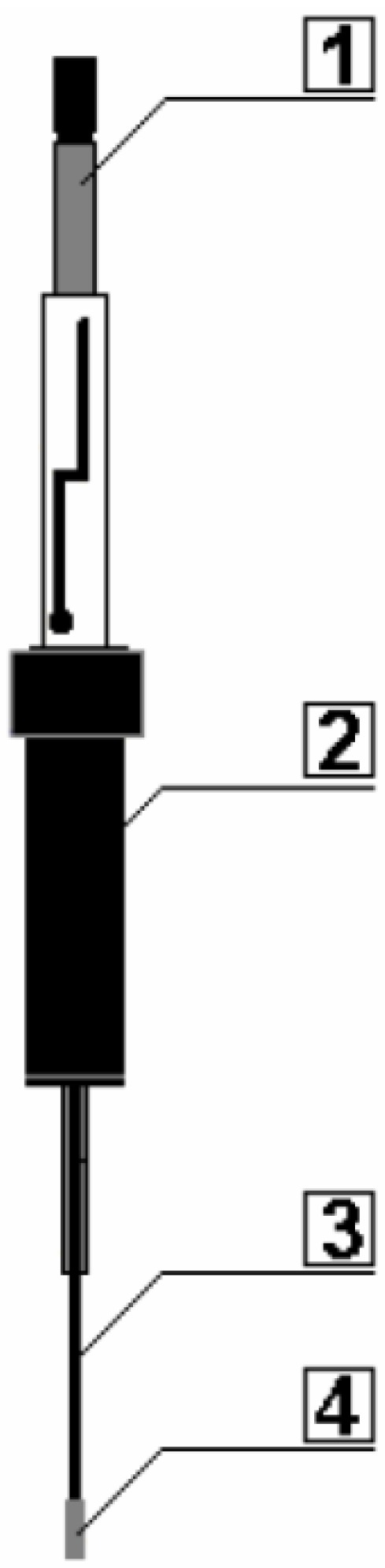
The structure of a stationary phase microextraction device (SPME). 1—piston; 2—cylinder; 3—needle; 4—extraction fibre.

The “heart” of the SPME system is the fibre (with a small diameter), which is made of fused silica and covered with an appropriate sorption material (extraction phase) installed in the microsyringe for protection and ease of manipulation (Figure 8). The fibre can be slid in or out of the syringe needle. The syringe needle is used for puncturing the divide during sample extraction and the desorption operation in a convenient manner. When the fibre is exposed directly in the tested aqueous sample (DI-SPME) or during its headspace phase (HS-SPME) [58], a phenomenon of analyte division occurs between the aqueous phase (the matrix) and the stationary phase placed on the SPME fibre. The SPME slid into the syringe needle is introduced into the dispenser of a liquid [57,59] or gas [57,60] chromatograph where, after sliding out, the analytes are desorbed and transferred to the chromatographic column using a carrier gas to separate them and then to the appropriate detector [27,52,55].

The SPME set is usually used in combination with gas chromatography (GC), high-performance liquid chromatography (HPLC) [59,61,62,63]; it is more rarely coupled with a set for capillary electrophoresis (CE) [61,64,65], and supercritical septum fluid chromatography [57,66].

While considering the thermodynamic aspects of using an SPME set, it can be concluded that the number of analytes extracted by the fibre is directly proportional to the concentration of analytes in the sample and independent of the fibre position (in the sample or in the stationary phase) [57].

The selection of a suitable extraction fibre is very important as the type of the stationary phase covering the core of the fibre influences the efficiency and selectivity of the solid-phase microextraction technique [52,61,67,68]. The choice of the polymer type used for the extraction is dependent on the chemical nature of the analyte such as polarity or volatility. In general, the polar fibres are used for polar analytes extraction, and the non-polar fibres for the non-polar analytes extraction. Polydimethylsiloxane is the most suitable type of liquid coating. It has found application in various analytical procedures using the SPME technique for the organometallic compounds analysis. Table 3 presents information about characteristics of commercially available fibres of the SPME device (PDMS and others), and their applicability not only with regard to the different forms of mercury and tin, but also other xenobiotics present in environmental samples.

The optimally selected type of the extraction fibre cover ensures:

- good reproducibility;
- low numerical values of the level of detection;
- reduction of the extraction time;
- reduction of the number of extracted impurities, which, as a result, allows for obtaining chromatograms of considerably better quality.

The thickness of the stationary layer has a significant influence on the extraction parameters. The application of a large amount of the stationary phase influences:

- the prolongation of the time to achieve the state of equilibrium [52,57];
- extended desorption rate;
- incomplete desorption—memory effect.

Therefore, this variant is used for extraction of volatile compounds.

The use of fibres with a thin film of the stationary phase ensures quick diffusion and easy release (during thermal desorption) of compounds, which, in turn, allows for isolating and enriching compounds characterised by a high boiling point [1,52,56].

In the analytical procedures regarding the determination of the different forms of mercury and tin in environmental samples using the SPME technique different types of fibre extraction/different substances covering fibre are used. Thus, the total efficiency of the extraction depends on both the type of fibre, the film thickness and the substance (the degree of volatility). Table 2 summarizes the literature published data on the application of different fibre types, the use of different film thickness, and other parameters, in the procedures of tin, and mercury speciation using the SPME technique.

**Table 3:** Characteristics of extraction fibres for commercially available SPME set [27,29,52,57].

Fibre Cover	Acronym	Thickness of the Film (µm)	Final Determination	Application
of Fibre with Non-polar Cover				
Polydimethylsiloxane	PDMS	100 30 7	GC, HPLC	Non-polar organic compounds (Hg^0^, MetHg, MBT, DBT, TBT, MPhT, DPhT, TPhT), VOCs, PAHs, BTEX
Fibre with Polar Cover				
Polyacrylate	PA	85	GC, HPLC	Polar organic compounds, triazine, phosphorganic, pesticides and phenols
Fibres with Mixed-Properties Cover				
Polydimethylsiloxane‒Polydivinylbenzene	PDMS-DVB	65 60 GC	HPLC	Aromatic hydrocarbons aromatic amines, VOCs, TMT, DMT, MMT, MBT, DBT, TBT
Polydimethylsiloxane‒Carboxen	PDMS-CAR	75	GC	Gaseous/volatile analytes (Hg^0^, MeHg), VOCs, hydrocarbons
Carbowax‒Polydivinylbenzene	CW-DVB	65	GC	Polar organic compounds, alcohols, ketones, nitroaromatic compounds
Carbowax-resin with molecular print	CW-TPR	50	HPLC	Anion surfactants, aromatic amines
Polydimethylsiloxane‒Polydivinylbenzene/Carboxen	PDMS/DVB/CAR	50/30	GC	Hg^0^, MeHg, DBT, TBT

The efficiency of the extraction process can also be modified by transferring analytes into derivatives [52,61]. The main parameters influencing the liquid and stationary phase microextraction processes are presented in Table 4.

**Table 4:** Parameters affecting the efficiency of the microextraction processes.

Technique	Parameters	References
LPME	- type of extraction solvent - type of dispersing solvent - volume of the extracting agent - volume of the dispersing agent - volume of the sample - mixing intensity - extraction time - salting out - pH of the sample	[11,12,13,26,69]
SPME	- extraction conditions (temperature, extraction time, mixing method) - ionic strength of solutions - stationary phase volume - headspace phase volume - volume of the sample - pH of the sample - using additions (salt or solvent) - type of material from which the fibre is made	[52]

The derivatisation process is usually used at the sample collection stage while determining polar or thermally unstable compounds in the SPME technique. The transformation of these compounds into more volatile derivatives allows for easier/faster/more effective extraction and enables the reduction of the limits of detection by as much as three orders of magnitude [29,70]. The combination of SPME and alkylborate reagents was used for the derivatisation of:

- inorganic and organic forms of lead [30,34,71,72,73,74,75,76];
- organic forms of mercury [34,71,74,77];
- organic forms of tin [32,34,40,71,74,77,78].

In SPME, derivatisation can be conducted in three different modes:

(1) simultaneously with SPME sampling;
(2) after the analyte is already in the fibre;
(3) when the analyte is desorbed to GC (in the dispenser) [29,79].

## 5. Directions for the Development and Possibilities of Using Microextraction Techniques

Various types of human activity (anthropopressure) are connected with emissions of considerable amounts of mercury and tin, as well as other heavy metals, into the environment. In view of the fact that these xenobiotics are characterised by the fact that their toxicity, mobility, bioavailability and bioaccumulation depends on the chemical form, it is necessary to determine the individual forms of metals and not their total concentrations in samples. This type is possible if speciation analysis is used, for which microextraction is very often employed at the sample preparation stage [29]. Table 5 presents the advantages and disadvantages of selected microextraction techniques. Table 2, on the other hand, presents literature information about the possibility of using selected microextraction techniques for determining various forms of tin and mercury.

Microextraction techniques are more and more often used in analytical procedures intended for tests of environmental samples, food, medication, as well as samples of biological origin.

In many environments, works are conducted, which are aimed at:

- modifying already known methodological solutions;
- developing new variants of such techniques, which are characterised by better metrological parameters.

**Table 5:** Advantages and disadvantages of selected microextraction techniques.

Advantages		Disadvantages	Technique	References
Cheap Easy to use little use of the solvent		Impermanence of drops Low sensitivity and precision	SDME	[11]
Cheap Easy to use Quick High flexibility in the selection of operating parameters (e.g., amount of the solvent, mixing speed)		Impermanence of drops Low sensitivity and precision Limited solvent choice	DI-SDME	[11,12,13,14]
Possibility of using various solvents Excellent cleaning of samples with a complex matrix composition Possibility of extracting volatile and water-soluble analytes Easy to use Quick Cheap		Low sensitivity and precision	HS-SDME	[13]
High repeatability Good clean-up ability High numerical value of the enrichment coefficient Cheap Easy to automate and miniaturise		Relatively long extraction time Possibility of fibre pores getting blocked	HF-LPME	[11]
Cheap Quick Requires the use of a small amount of sample Requires the use of a small amount of organic solvents High numerical value of the enrichment coefficient The extraction efficiency does not depend on the time The balance time is established over a very short period of time High recovery		Low selectivity Requires the use of three solvents Limited solvent choice Requires centrifugation Not appropriate for samples with a complex matrix composition	DLLME	[11,13]
Requires the use of a small amount of organic solvents Easy to use High numerical value of the enrichment coefficient	-		LLLME	[15]
Quick Cheap Low analyte losses Easy to use Possibility of implementing an analytical procedure on-line Possibility of using liquid, gaseous and solid samples, High sensitivity, Easy to automate and miniaturize	Relatively expensive (fibre cost) Limited time of fibre use Matrix effects Fibre damage		SPME	[27,29,52,71]
Continuous contact between the solvent drop and a fresh sample solution Possibility of accurate control of the solvent drop size (combined with HPLC) High numerical value of the enrichment coefficient Requires the use of a small amount of sample	-		CFME	[23]

An example can be continuous-flow microextraction. In this technique, just like in the other microextraction techniques, modifications aimed at extending its scope of application have been introduced. Modifications and improvements of this technique allowed to use it in the analytical procedures for the determination of metals in biological and environmental samples. Thus, the new method—IL-based cycle flow SDME combined with ETV-ICP-MS was used for the determination of mercury-containing samples [80,81].

Some of the major areas of concern in terms of innovation and new solutions of microextraction techniques include the use of:

- electromembrane HF(3)ME extraction (EME) of ionized species;

In this method, an electrical potential applied across the SLM is used as driving force for creating the flux of uncharged analytes towards the acceptor phase. Furthermore, the extraction is assisted by strong stirring in order to reduce the standing (stagnant) organic layer near the SLM and to induce convection in the sample. After aqueous acceptor extraction, the solution may be transferred directly into the HPLC and CE [13].

At the moment knowledge about EME is limited, and therefore, still more research is carried out to understand the exact extraction mechanism in order to optimise specific analyte extraction conditions/parameters, and to demonstrate that this technique provides reliable data for a wide range of analytical applications. The electrokinetic membrane extraction is an interesting concept which in the future will allow the sample preparation to be integrated with separation techniques [13].

- SME procedures for practical on-line sample analyses;
- solvents less dense than water in DLLME;
- ionic liquids (ILs);
- ultrasound-assisted emulsification for DLLME [19].

Ionic liquids are becoming more popular due to the low consumption of volatile organic solvents and a high rate of enrichment thanks to the use of ultrasound; certain stages of the analytical procedure such as homogenizing, emulsifying or extraction can be accelerated. The combination of ionic liquids and the dispersion by sonication of a liquid–liquid microextraction allowed to design a new method for the extraction—IL-USA-DLLME—which is as fast, simple and effective as the DLLME technique, but does not require the use of organic solvents. Until now, this method “was proven” during the determination of cadmium, mercury or phytocides, but it also has the potential for the determination of trace amounts of other metal ions in samples with complex matrices [82,83]. However, not all of these new approaches/solutions have been recognised in the determination of mercury and tin compounds.

New trends pertaining to research on improving microextraction techniques concentrate on: the use of new covers characterised by higher extraction efficiency, higher selectivity and durability, the development of new derivatisation and calibration methods.

## 6. Conclusions

The presence of metalorganic compounds in all elements of the environment has increased over the past decades in connection with human activity [39]. Toxicity, biochemical properties and the mercury and tin cycle in the environment depends on the concentration and chemical form of these elements. The determination of organomercury and organotin compounds, MeHg, DMeHg, TBT, DBT, is particularly important due to their high toxicity, teratogenicity and carcinogenicity. As a result, it is necessary to continuously monitor the xenobiotic content levels in the individual elements of the environment using analytical procedures characterised by appropriate metrological parameters:

- high precision and accuracy;
- low numerical value of parameters, such as: the limit of quantification and the value of detection;
- high selectivity [84].

Due to the low levels of the content of chemical forms of tin and mercury in some environmental and biological samples in appropriate analytical procedures, it is necessary to use the separation and enrichment stage before the analysis of appropriately prepared samples [15]. Traditional extraction methods are usually time-consuming, they require the use of multi-stage sample preparation processes and the use of large quantities of organic solvents, there is also a risk of analyte loss [27]. For this reason, more emphasis is placed on compliance with “green chemistry” principles; therefore, the use of large quantities of organic solvents in analytical laboratories is not tolerated any more, due to the related hazard to the health of living organisms. As a result, over the past several years, numerous solvent-free extraction methods, as well as other methods with low solvent consumption have been discovered and described [27,51], which is why microextraction techniques are used both for analyte extraction and their derivatisation. However, analytical chemists are still looking for new methodological and instrumental solutions, which, when used at the stage of preparing samples for analysis, make analytical procedures meet the expectations of analysts and will provide an opportunity for obtaining measurable results containing the levels of metalorganic compound levels in representative samples and processes, which these analytes are subjected to in the individual elements of the environment.

## List of abbreviations and acronyms

Abbreviation Acronym	Explanations
LPME	Liquid-Phase Microextraction
LLE	Liquid-Liquid Extraction
HS-SDME	Headspace Single-Drop Microextraction
SDME	Single-Drop microextraction
DLLME	Dispersive Liquid-Liquid Microextraction
HF-LPME	Hollow-fibre liquid-phase microextraction
DI-SDME	Direct Immersion Single-Drop Microextraction
LLLME	Liquid-Liquid-Liquid Microextraction
CFME	Continuous flow microextraction
ETV-ICP-MS	Electrothermal vaporisation Inductively Coupled Plasma Mass Spectrometry
CPE	Cloud point extraction
GC	Gas Chromatography
HPLC	High-performance liquid chromatography
CE	Capillary Electrophoresis
AS	Atomic Spectroscopy
SPME	Solid-Phase Microextraction
SPE	Solid-Phase Extraction
DI-SPME	Direct Immersion Solid-Phase Microextraction
HS-SPME	Headspace Solid-Phase Microextraction
MS	Mass Spectrometry
ICP	Inductively Coupled Plasma
HS-LPME	Headspace Liquid-Phase Microextraction
LLLPME	Liquid–Liquid–Liquid-Phase Microextraction
EME	Electrokinetic membrane extraction
